# Response Surface Methodology Optimization of Time-Resolved Fluorescence Immunoassay for Rapid Detection of AflatoxinB_1_ in Yellow Rice Wine

**DOI:** 10.3390/toxins17050248

**Published:** 2025-05-16

**Authors:** Mengjie Zhu, Dun Wang, Du Wang, Jing Dong, Xue Wang, Qi Zhang, Man Xiao

**Affiliations:** 1 Xiangyang Academy of Agricultural Sciences, Xiangyang 441057, China; zmj2280880980@163.com (M.Z.); xynkydongjing@163.com (J.D.); maysxuer@126.com (X.W.); 2 Oil Crops Research Institute, Chinese Academy of Agricultural Sciences, Wuhan 430062, China; wang416929@126.com; 3 College of Food Science and Engineering, Wuhan Polytechnic University, Wuhan 430023, China; 18245361699@163.com

**Keywords:** response surface design, rapid detection, aflatoxinB_1_, yellow rice wine, time-resolved fluorescence immunoassay, optimization

## Abstract

Yellow rice wine is susceptible to aflatoxinB_1_ (AFB_1_) contamination, yet existing detection technologies suffer from limitations such as high false-positive rates, cumbersome operational protocols, or elevated costs, rendering them inadequate for large-scale screening requirements. Consequently, the development of a highly sensitive and rapid detection method for AFB_1_ is urgently needed to provide technical support for quality supervision and risk assessment of yellow rice wine. In this study, AFB_1_ detection was performed using time-resolved fluorescence immunoassay technology, with quantitative analysis based on the ratio of the T signal value of the detection line to the C signal value of the quality control line and the natural logarithmic value of the standard solution concentration. Statistical experimental designs were used to optimize the process of this rapid detection of AFB_1_ in yellow rice wine. The most important factors influencing recovery rate (*p* < 0.05), as identified by a two-level Plackett-Burman design with 11 variables, were methanol-water volume fraction, sample to extraction solvent ratio, heating temperature, and heating time. The steepest ascent method was employed to identify the optimal regions for these four key factors. Central composite design (CCD) coupled with response surface methodology (RSM) was subsequently utilized to further explore the interactive effects among variables and determine their optimal values that maximize the recovery rate. The analysis results indicated that interactions between methanol-water volume fraction and other three factors–sample to extraction solvent ratio, heating temperature, heating time–affected the response variable (recovery rate) significantly. The predicted results showed that the maximum recovery rate of AFB_1_ (97.35%) could be obtained under the optimum conditions of a methanol-water volume fraction of 78%, a sample to extraction solvent ratio of 1:3.2, a heating temperature of 34 °C, and a heating time of 6.4 min. These predicted values were further verified by validation experiments. The excellent correlation between predicted and experimental values confirmed the validity and practicability of this statistical optimum strategy. Optimal conditions obtained in this experiment laid a good foundation for further use of time-resolved fluorescence immunoassay for rapid detection of AFB_1_ in yellow rice wine, demonstrating broad application prospects.

## 1. Introduction

As a traditional fermented alcoholic beverage in China, yellow rice wine holds an important position among the world’s major fermented alcoholic products (such as beer, sake, etc.) [1]. Yellow rice wine products have distinct national characteristics and industrial advantages. With a unique flavor, they are rich in active substances such as proteins, amino acids, and polypeptides. Moderate consumption of yellow rice wine has certain nutritional value and health benefits for the human body [2]. However, research on the metabolic mechanisms of harmful substances in yellow rice wine and the control technologies for these substances started relatively late, compared with beers and wines. There is a lack of mature research findings and experiences that can be drawn upon from abroad [3]. Fungal toxins, as secondary metabolites of certain microorganisms, have always been a safety concern in the food industry. AFB_1_ is an extremely toxic and highly carcinogenic substance that easily contaminates grains, nuts, groundnuts, dried fruits, spices, and other food products. It has a high melting point (265–269 °C), and its crystalline state remains relatively stable under high-temperature conditions [4,5,6]. As an ingredient in the production of yellow rice wine, raw wheat koji is made from raw wheat. The process involves crushing the wheat, mixing it with water, and directly forming it into blocks. It is then naturally enriched and cultured in a natural environment relying on wild microorganisms attached to the surface of the wheat (such as *Absidia corymbifera*, *Aspergillus oryzae*, *Rhizopus*, *lactic acid bacteria*, etc.). However, yellow rice wine factories in different regions often produce their own koji. The climates, environments, and koji room conditions vary significantly across these regions, leading to substantial differences in the microbial composition of the koji. *Aspergillus flavus*, which produces aflatoxin, is highly likely to proliferate [7]. Moreover, most yellow wine brewing processes are conducted in an open, non-sterilized manner. Various raw materials and tools used in the process are heavily contaminated with microorganisms. Both beneficial and harmful microorganisms present in the brewing environment and air have the opportunity to infiltrate [8,9,10]. Studies have shown that the levels of AFB_1_ in various samples during the production process of distilled spirits (including daqu, fermented mash, spent grains, base liquor, and finished liquor) are far below 5 μg·kg^−1^. In particular, AFB_1_ is almost non-existent in base liquor and finished liquor. During the yellow rice wine brewing process, both the mash and the yellow wine itself are relatively nutrient-rich substrates. Although the low pH, alcohol content, and mash concentration during production exert some inhibitory effects on microorganisms, unlike distilled spirits that undergo a distillation process, some non-cultivable microbial species may still proliferate, this increases the possibility of AFB_1_ contamination [11]. Ji Xiaofeng et al. [12] found that in the 26 bottled yellow wine samples tested, although the content of AFB_1_ did not exceed the maximum limit for fermented foods (5 μg·kg^−1^), the detection rate was as high as 100%. Moreover, AFB_1_ is highly stable under normal conditions, especially in alcoholic beverages. Once formed, it is extremely stable and difficult to degrade. Thus, with the rapid growth of the yellow wine consumer market and the increasing awareness of nutrition, health, and safety among consumers, the quality and safety issue of AFB_1_ in yellow wine is also drawing greater attention from consumers. Therefore, it is urgent and necessary to establish highly sensitive and rapid detection methods for AFB_1_.

The current detection methods for AFB1 in yellow wine mainly include instrumental analysis, enzyme-linked immunosorbent assay (ELISA), and fluorometry techniques. Among these, ELISA is the most commonly used. However, due to the possibility of false-positive results, it is generally only used for preliminary screening. Instrumental analysis methods such as HPLC and LC-MS/MS have improved the accuracy and sensitivity of analysis. However, their disadvantages, such as high equipment costs, the need for professional technicians, and high detection costs, make them unsuitable for large-scale sample testing [13,14]. Traditional fluorometry techniques have been limited in development due to issues such as environmental pollution and significant background fluorescence interference. Compared with fluorescence-based methods, fluorescence immunoassay (FIA) is used to quantify specific molecules such as proteins or cytokines as they rely on target recognition and binding by specific antibodies that have been labeled with a fluorophore. This high specificity makes immunological analysis highly reliable and accurate when detecting complex samples, and it can also detect target molecules at extremely low concentrations. Hu Zhenzhen et al. [15] used cationic polymers to cause quantum dot aggregation and fluorescence quenching, and with the help of alkaline phosphatase (ALP) catalyzing the removal of phosphate groups from DNA, the quantum dots were released, and fluorescence was restored. This method can detect ALP at concentrations as low as 0.1 mU/mL. Zhang Wenping et al. [16] induced the aggregation of the perylene derivative (Probe-1) with polyphosphoric acid (PPA) to quench fluorescence. This method has a detection limit of 0.5 mU/mL and is easy to operate, cost-effective, and highly selective. However, the primary limitation of conventional FIA is the pronounced background interference stemming from the intricate matrices of co-existing luminescent substances in food and feed. Consequently, mitigating background interference is of utmost importance for achieving highly sensitive determinations. Time-resolved fluorescence immunoassay (TRFIA) uses trivalent rare-earth ions that emit fluorescence, such as europium (III), terbium (III), and samarium (III), as labeling agents. Because the fluorescence lifetime of specific target signals is several orders of magnitude longer than that of non-specific background noises, as the detection time elapses, background signals with short-life can be eliminated, while the fluorescent lanthanide chelates with long-life (Eu^3+^, Tb^3+^, and Sm^3+^, etc.) can afford an effective and reliable proportion of fluorescence signals to the contents of the sample. As a result, TRFIA offers advantages such as high sensitivity, strong resistance to matrix interference, and robust stability [17,18,19,20].

However, as far as we know, there is limited knowledge about time-resolved fluorescence immunoassay for rapid detection of AFB_1_ in yellow rice wine. Therefore, it is necessary to design an appropriate process for maximizing the recovery rate. Statistical experimental designs such as Plackett-Burman (PB) and response surface methodology (RSM) [21] can collectively optimize all the affecting parameters to eliminate the limitations of a single-factor optimization process. The PB design offers a rapid and efficient approach to screen significant factors from a large pool of variables, thereby optimizing resource allocation while preserving robust statistical information for each parameter [22]. Response surface methodology (RSM), integrating factorial design, and regression analysis, facilitates the evaluation of critical factors, construction of predictive models to investigate variable interactions, and selection of optimal conditions to achieve desired responses [23].

In the present study, a Plackett-Burman design has been employed to determine the significant factors affecting recovery rate. A steepest ascent method and a central composite experimental design (CCD) were used to identify the optimal levels of significant factors to enhance the recovery rate by time-resolved fluorescence immunoassay for rapid detection of AFB_1_ in yellow rice wine.

## 2. Results and Discussion

### 2.1. Method Detection Line and Linear Range

**Figure 1:**
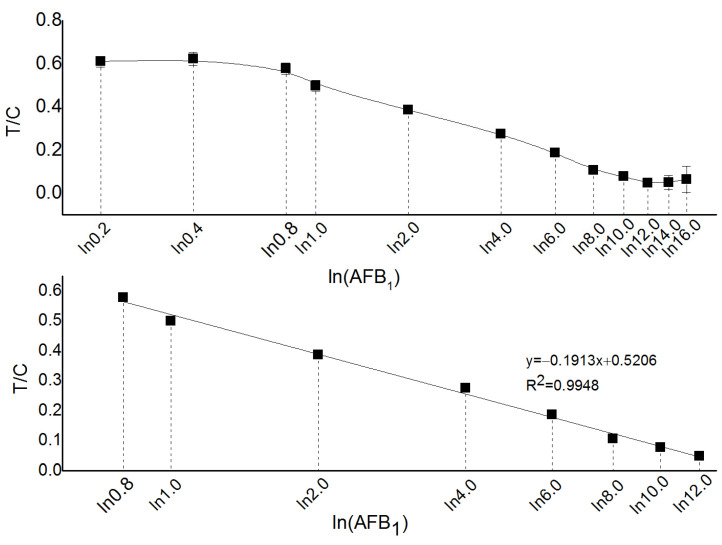
AFB_1_ dose-response curve in yellow wine. (T/C represents the ratio of the T signal value of the detection line to the C signal value of the quality control line, In(AFB_1_) represents the natural logarithmic value of the AFB_1_ standard solution concentration).

The method detection line and linear range are shown in Figure 1. When the content of AFB_1_ is less than 0.8 µg·kg^−1^ or greater than 12.0 µg·kg^−1^, its concentration shows a non-linear correlation with the test strip, and the experimental results have larger errors. Therefore, the linear detection range of the time-resolved fluorescence immunoassay method for AFB_1_ in yellow rice wine is 0.8–12.0 µg·kg^−1^. The corresponding linear equation of the standard curve is y = −0.1913x + 0.5206 (R^2^ = 0.9948), with a standard deviation of 0.018. The method limit of detection (LOD) can be calculated as 0.3 µg·kg^−1^ according to Equation (1).

LOD = 3S_b_/b (1)

where LOD represents the limit of detection; S_b_ represents the standard deviation of the blank value; b represents the slope of the method calibration curve.

### 2.2. Results of the Single-Factor Experiment

**Figure 2:**
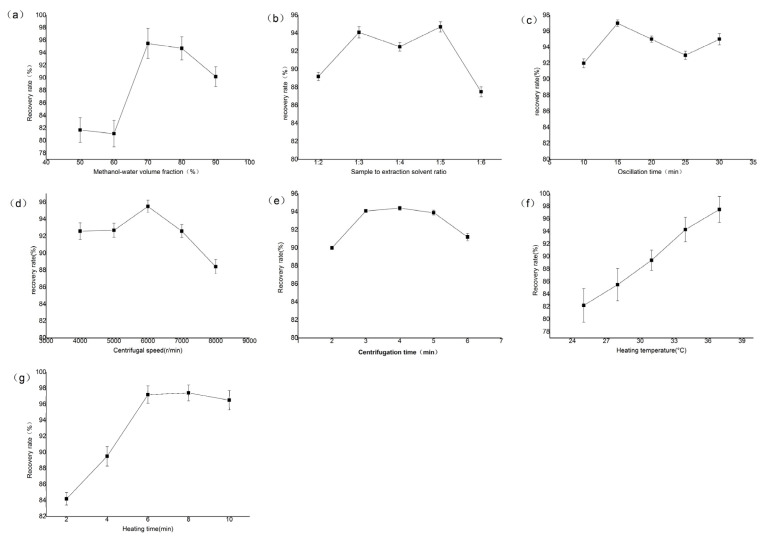
Results of a single-factor experiment: (**a**) methanol-water volume fraction; (**b**) sample to extraction solvent ratio; (**c**) oscillation time; (**d**) centrifugal speed; (**e**) centrifugation time; (**f**) heating temperature; (**g**) heating time.

The results of the single-factor experiment are shown in Figure 2. As shown in Figure 2a, the recovery rate first increased and then decreased with the increasing volume fraction of methanol-water solvent. This trend may be attributed to the fact that AFB_1_ is poorly soluble in water. Therefore, the extraction amount is low when the volume fraction of the organic solvent is small. As the volume fraction of the organic solvent increases, the solubility of lipophilic components in yellow rice wine also increases, and these components may affect the solubility of AFB_1_, resulting in a lower extraction amount. Thus, methanol-water volume fraction of 70% is considered as the optimized range for further PB experimental trials.

As shown in Figure 2b, the recovery rate first increased and then decreased with the increase in the sample to extraction solvent ratio. This trend may be due to the fact that, as the contact area between yellow rice wine and the solvent increases and the solvent concentration rises, the solubility of AFB_1_ in the sample is enhanced. Once the solvent reaches a certain concentration, it becomes saturated, and the solubility of AFB_1_ tends to stabilize, resulting in little change in the recovery rate. Further increasing the sample-to-extraction solvent ratio beyond this point may lead to the dissolution of other impurities in the sample, thereby reducing the recovery rate. Therefore, a sample to extraction solvent ratio of 1:3 was selected for the PB experiment.

As shown in Figure 2c,g, the recovery rate of AFB_1_ exceeded 90% when the oscillation time was greater than 15 min and the heating time was greater than 6 min. Oscillation and heating are beneficial for the dissolution of aflatoxins and the binding of antigens and antibodies. Further increasing these times resulted in minimal changes in the recovery rate. Based on the principle of cost-saving, an oscillation time of 15 min and a heating time of 6 min were selected for the PB experiment.

As shown in Figure 2d,e, the recovery rate first increased and then decreased with the increase in centrifugation speed and centrifugation time. To some extent, the higher the centrifugation speed and the longer the time, the higher the extraction rate of AFB_1_. However, when increased beyond a certain level, it may lead to the dissolution of impurities, resulting in a decrease in the extraction amount and thus affecting the recovery rate of AFB_1_. Therefore, a centrifugation speed of 6000 r/min and a centrifugation time of 4 min were considered for the PB experiment.

As shown in Figure 2f, the recovery rate of AFB_1_ gradually increased with the rise in extraction temperature. To some extent, increasing the temperature is conducive to the binding of antigen and antibody. However, considering that antibodies may denature at temperatures above 37 °C, a heating temperature of 37 °C was selected for the PB experiment.

### 2.3. Screening of Important Variables Using Plackett-Burman Design

A Plackett-Burman design with 12 runs was applied to evaluate eleven factors (including four dummy variables). Each variable was tested at two levels. Table 1 presents the variables and their corresponding levels used in the experimental design. The Plackett-Burman experimental design and the response values of AFB_1_ recovery rate are shown in Table 2.

The data listed in Table 3 indicated a wide variation in recovery rate, from 72.5% to 94.8%, in the 12 trials. The variation suggested that process optimization was important for improving the removal efficiency of recovery rate. The analysis of the regression coefficients and *p*-values of the seven factors in Table 3 indicates that the Model F-value of 38.97 implies the model is significant. There is only a 0.16% chance that an F-value this large could occur due to noise.

Variables with a confidence level exceeding 95% are considered significant parameters (*), those with a confidence level exceeding 99% are considered highly significant parameters (**), and those with a confidence level exceeding 99.9% are considered extremely significant parameters (***). It is evident that variable B is a significant factor, variable G is a highly significant factor, and variables A and F are extremely significant factors. In contrast, variables C, D, and E, with confidence levels below 95%, are deemed insignificant and were therefore not included in the subsequent steepest ascent and central composite design (CCD) experiments. The model equation for the recovery rate (Y) can be written as:

Y = 85.99 + 4.73A + 1.56B − 1.23C + 0.025D + 0.475E + 4.26F + 3.06G(2)

### 2.4. Path of Steepest Ascent

Tested variables (Methanol-water volume fraction, sample to extraction solvent ratio, heating temperature, heating time) were denoted as X_l_, X_2_, X_3_, and X_4,_ respectively. The path of steepest ascent was based on the zero level of the Plackett-Burman design and moved along the direction in which methanol-water volume fraction, sample to extraction solvent ratio, heating temperature, and heating time increased. The experimental design and results are shown in Table 4. The highest response was 96.1% with methanol-water volume fraction of 75%, a sample to extraction solvent ratio of 1:3.2 (V/V), a heating temperature of 33 °C, and a heating time of 6 min. This point was concluded to be near the optimal point and was chosen for further optimization.

### 2.5. Optimization by Response Surface Methodology

#### 2.5.1. RSM Regression Equation and Model Analysis

Central composite design (CCD) was utilized to investigate the interactions among significant factors and to determine their optimal levels. In this study, a four-factor, five-level CCD with 30 runs was employed. Tested variables were assessed at five different levels, combining factorial points (−1, +1), axial points (−2, +2), and central point (0), as shown in Table 5.

The design matrix of tested variables and the experimental results are represented in Table 6. The second-order model used to fit the response to the independent variables is shown in Equation (3):

(3) $$Y=\beta_{o}+\sum_{i=1}^{k}\beta_{i}x_{i}+\sum_{i=1}^{k}\beta_{ii}x_{i}x_{i}+\sum_{1\ll i\ll j}^{k}\beta_{ij}x_{i}x_{j}$$

where Y is the predicted response (recovery rate); Xi and Xj are input variables that influence the response Y; k is the number of variables; β_0_ is the constant term; β_i_ is the ith linear coefficient; β_ii_ is the iith quadratic coefficient, and β_ij_ is the ijth interaction coefficient.

The model’s adequacy was evaluated using analysis of variance (ANOVA), as presented in Table 7. Multivariate regression analysis was performed to derive the following second-order polynomial equation:

Y = 95.67 + 2.55X_1_ + 0.9625X_2_ + 2.01X_3_ + 1.83X_4_ + 0.8688X_1_X_2_ + 1.04X_1_X_3_ + 2.16X_1_X_4_ − 1.66X_2_X_3_−0.3937X_2_X_4_ + 0.5313X_3_X_4_ − 3.09X_1_^2^ − 2.88X_2_^2^ − 2.98X_3_^3^ − 4.17X_4_^2^ (4)

The Model F-value of 121.96 implies the model is significant. There is only a 0.01% chance that an F-value this large could occur due to noise.

*p*-values less than 0.0500 indicate model terms are significant. In this case X_1_, X_2_, X_3_, X_4_, X_1_X_2_, X_1_X_3_, X_1_X_4_, X_2_X_3_, X_3_X_4_, X_1_^2^, X_2_^2^, X_3_^2^, X_4_^2^ are significant model terms. The coefficient of determination (R^2^) was calculated as 0.9918 for recovery rate, indicating good agreement between the experimental and the predicted values. The pred-R^2^ of 0.9584 was in reasonable agreement with adj-R^2^ of 0.9832.

The Lack of Fit F-value of 1.70 implies the Lack of Fit is not significant relative to the pure error. There is a 28.93% chance that a Lack of Fit F-value this large could occur due to noise. Non-significant Lack of Fit is good—we want the model to fit. The model was found to be adequate for prediction within the range of variables employed.

#### 2.5.2. Mutual Interactions Between the Significant Factors

**Figure 3:**
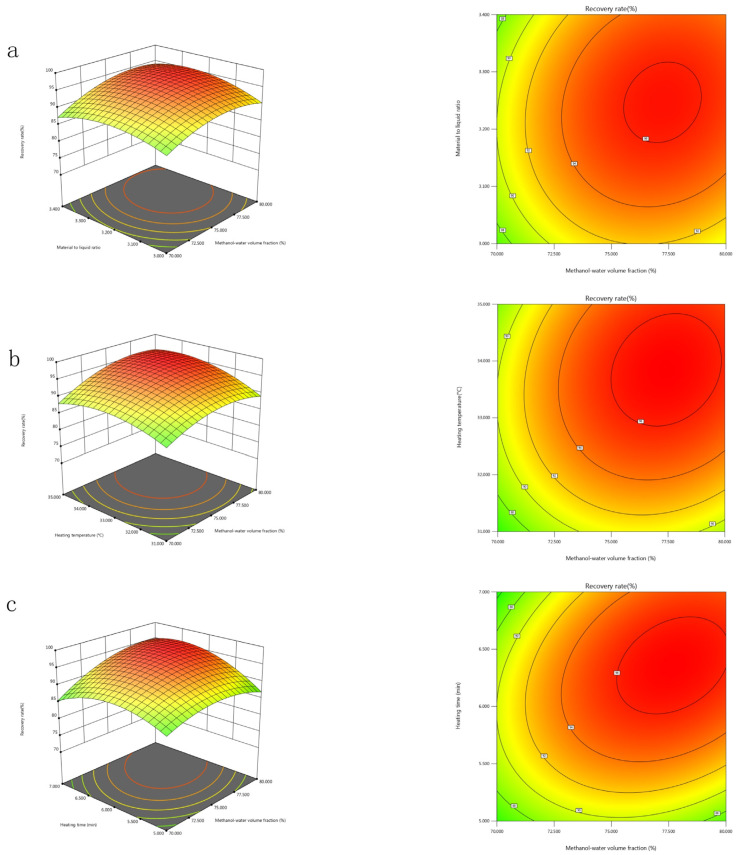
Three-dimensional response surface plots and two-dimensional contour plots for recovery rate showing variable interactions of: (**a**) methanol-water volume fraction and sample to extraction solvent ratio; (**b**) methanol-water volume fraction and heating temperature; (**c**) methanol-water volume fraction and heating time.

**Figure 4:**
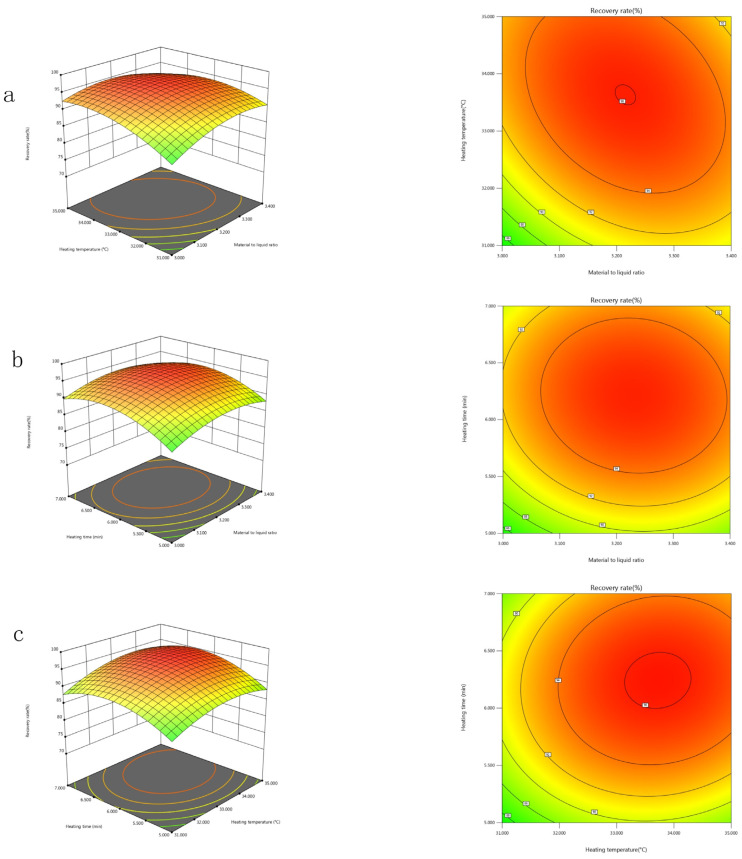
Three-dimensional response surface plots and two-dimensional contour plots for recovery rate showing variable interactions of: (**a**) sample to extraction solvent ratio and heating temperature; (**b**) sample to extraction solvent ratio and heating time; (**c**) heating temperature and heating time.

The regression model was visualized using response surface plots and corresponding contour plots generated by Design-Expert Version 13 (Stat-Ease Ine., Minneapolis, MN, USA), as depicted in Figure 3 and Figure 4. Each response surface plot illustrates the interactive effects of two independent variables while maintaining the remaining variables at their zero levels. The contour plot shapes reveal the significance of interactions between independent variables. As shown in Figure 3 and Figure 4, each response surface of Y exhibits a distinct peak, indicating that the optimal point lies well within the design boundary.

**Figure 5:**
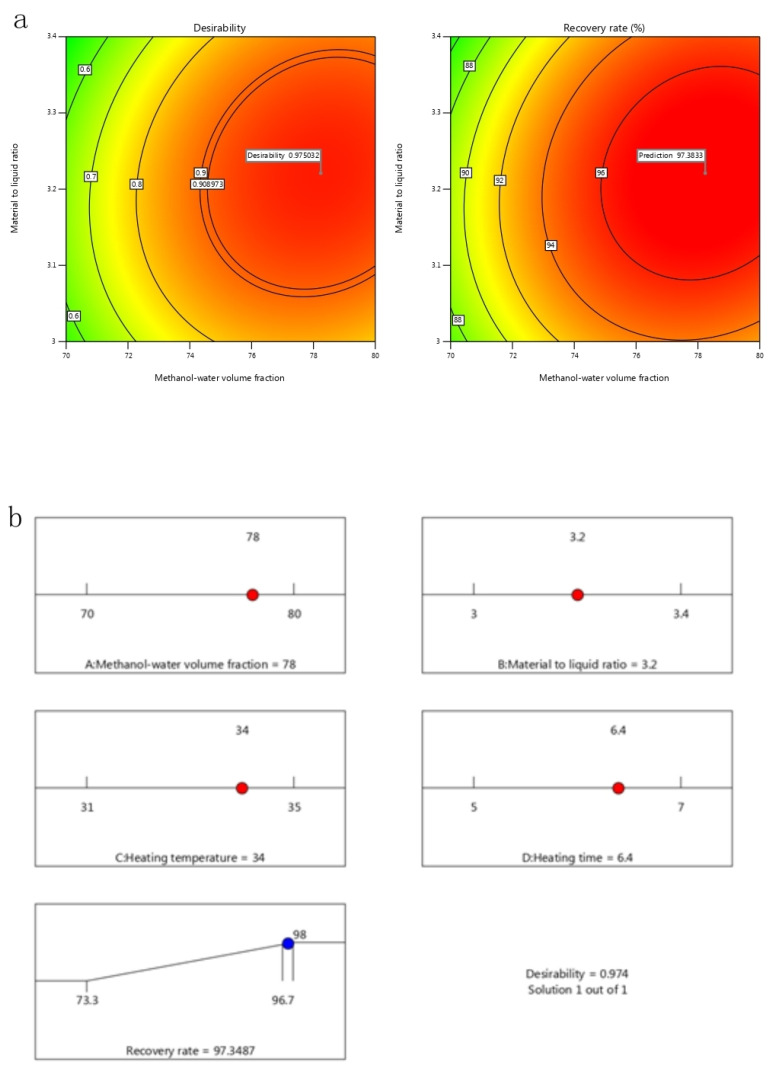
Contour of prediction of maximum recovery rate and its desirability: (**a**) at the optimal conditions; (**b**) at the optimized conditions.

According to the results of statistically designed experiments, optimal conditions were as follows: a methanol-water volume fraction of 78.238%, a sample to extraction solvent ratio of 1:3.221, a heating temperature of 33.918 °C, a heating time of 6.411 min, The predicted phenol degradation was 97.383%, and the predicted desirability was attained as 97.503%, as shown in Figure 5a. Considering practical feasibility, the experimental conditions are optimized as follows: methanol-water volume fraction of 78%, sample to extraction solvent ratio of 1:3.2, heating temperature of 34 °C, heating time of 6.4min. Under these optimized conditions, the predicted response for phenol degradation was 97.3487%, and the predicted desirability was attained as 98.95%, as shown in Figure 5b. The experimental value was quite close to the predicted value, which demonstrated the validity of the model. Therefore, response surface optimization could be successfully used to evaluate the performance in time-resolved fluorescence immunoassay for rapid detection of AFB_1_ in yellow rice wine.

### 2.6. Validation of the Model

#### 2.6.1. Intra-Assay Precision of the Method

Table 8 shows that the spiked recovery rates of the intra-assay of the method are 87.9–105.7%. As the labeled concentration and recovery rate increase, the Relative Standard Deviation(%RSD) of the measurement results decreases, primarily because smaller signal differences lead to greater impacts of signal interference on the results. With the %RSD ranging from 4.48% to 6.26%, it indicates that this detection technology exhibits good accuracy and precision of the intra-assay.

#### 2.6.2. Inter-Assay Precision of the Method

As shown in Table 9, the spiked recovery rates across different assays of the method range from 85.9% to 99.8%, with a %RSD between 6.10% and 8.86%. This demonstrates that the inter-batch accuracy and precision of this detection technology are favorable.

#### 2.6.3. Comparison of Result Between TRFIA and HPLC

**Figure 6:**
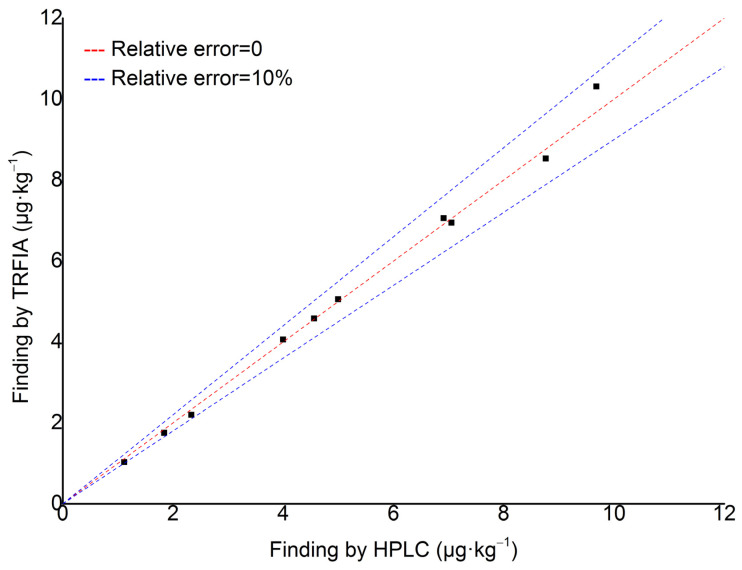
Comparison of detection results for spiked samples between TRFIA and HPLC.

As shown in Figure 6, for spiked samples, the time-resolved fluorescence immunoassay (TRFIA) developed in this study for the detection of AFB_1_ shows a relative error of less than 10% compared with the results obtained by the third method (high-performance liquid chromatography with post-column derivatization) in the national food safety standard (GB5009.22—2016) [24]. This indicates that the TRFIA technique for AFB_1_ detection is in good agreement with the national standard method.

But for natural samples, as shown in Table 10, for AFB_1_ content above the limit of quantitation (0.8 µg·kg^−1^), the relative error is less than 8%. However, when the AFB_1_ content is below the LOQ, the relative error may exceed 10%. It is particularly important to note that when the AFB_1_ content is below the limit of detection (LOD), TRFIA cannot detect AFB_1_ in yellow rice wine.

The results show that TRFIA has a good effect in the rapid detection of AFB_1_ in yellow rice wine. However, for trace detection of AFB_1_, it is still necessary to combine high sensitivity instruments such as HPLC and LC-MS/MS for analysis.

#### 2.6.4. Performance Comparison of TRFIA, ELISA, HPLC, and LC-MS/MS

As shown in Table 11, compared with the enzyme-linked immunosorbent assay (ELISA) method, the limit of detection (0.3 μg/L) and limit of quantitation (0.8 μg/L) of TRFIA are acceptable. However, typical potential matrix interferents (such as polyphenols, polysaccharides, and proteins) are present in yellow rice wine. ELISA operations involve color development and washing steps, making them relatively complex and the matrix interference is more significant. The time-resolved fluorescence immunoassay (TRFIA), grounded in antigen-antibody interactions, with a low antibody cross-reactivity rate [25], uses lanthanide elements (such as europium and samarium) to label antibodies. Through time-resolved technology, it separates background fluorescence, delays the detection of fluorescent signals, and eliminates interference from short-lived auto fluorescent substances (such as proteins and polyphenols) in yellow rice wine samples, resulting in higher specificity.

Although high-performance liquid chromatography (HPLC) and liquid chromatography-mass spectrometry/mass spectrometry (LC-MS/MS) exhibit extremely high sensitivity and a broad detection range, their applications are constrained by complex pretreatment and instrument parameter optimization—the entire analysis process takes 2–3 h. Therefore, these methods are more suitable for quantitative laboratory analysis of yellow rice wine or as complementary detection methods for rapid TRFIA screening.

## 3. Conclusions

As a key indicator for safety testing of yellow rice wine, the performance of detection methods for AFB_1_ is of utmost importance. This paper, for the first time, applies statistical experimental designs to optimize the method of time-resolved fluorescence immunoassay (TRFIA) for rapid detection of AFB_1_ in yellow rice wine.

Under the optimal conditions obtained in this experiment, the technique can complete batch screening within 30–60 min. It is highly suitable for production enterprises and grassroots inspection institutions to perform rapid detection of AFB_1_ in yellow rice wine, demonstrating broad application prospects.

## 4. Materials and Methods

### 4.1. Establish a Standard Curve

#### 4.1.1. Preparation of Sample Diluent

Weigh 1.0 g of sucrose, 0.5 g of bovine serum albumin, and 2.5 g of Tween 20 (from National Pharmaceutical Group Chemical Reagents Beijing Co., Ltd., Beijing, China), mix them thoroughly, and dilute to a final volume of 100.0 mL with ultrapure water (prepared using the UPH-III-10 type ultrapure water system) [28].

#### 4.1.2. Selection of Blank Matrix Solution

Select yellow rice wine samples with low impurities and a clear appearance (all purchased from large local supermarkets). The detection was carried out using the third method (high-performance liquid chromatography with post-column derivatization) specified in the National Food Safety Standard of China (GB 5009.22—2016). The instruments used included an HX-G type photochemical post-column derivatizer (from Wuhan Hengxin Reagent Technology Co., Ltd, Wuhan, Hubei Province, China) and a Shimadzu LC-20A liquid chromatograph. The column was ZORBAX Eclipse XDB-C18 (5 µm-Micron, 4.6 × 150 mm) bought from Agilent Technologies (China) Co., Ltd., Beijing, China, and the column temperature was 40 °C The mobile phase was Acetonitrile-methanol (50 + 50) and water (V:V = 32:68), and the flow rate was set as 1.0 mL/min. The detector was a fluorescence detector (excitation wavelength was 360 nm, emission wavelength was 440 nm). Under these conditions, the retention time for AFB_1_ is 6.195 min, and the detection limit is 0.02 µg·kg^1^. The total aflatoxin immunoaffinity column was sourced from Suweiwei Biotechnology Research Co., Ltd., Wuxi, Jiangsu Province, China. Samples that did not contain AFB_1_ were selected as the blank matrix solution.

#### 4.1.3. Standard Working Solution Preparation

AFB_1_ standard (2 mg·kg^−1^, from Beijing Tanmo Quality Inspection Technology Co., Ltd, Beijing, China) was dissolved in methanol (from Fisher, Waltham, Massachusetts, USA) and diluted to a final volume of 10 mL to prepare a standard stock solution with a mass concentration of 200 µg·kg^1^. The stock solution was stored at −20 °C for later use. The stock solution was then serially diluted with the blank matrix solution to prepare a series of standard working solutions with concentrations of 0.2, 0.4, 0.8, 1.0, 2.0, 4.0, 6.0, 8.0, 10.0, 12.0, 14.0, and 16.0 µg·kg^1^. These standard working solutions were used for time-resolved fluorescence immunoassay chromatographic detection of aflatoxin, from low to high concentrations. A standard curve was plotted based on the ratio of the test line signal to the control line signal (T/C) and the natural logarithm of the standard solution concentration (lnC) [28].

#### 4.1.4. Detection Limit and Linear Range

The detection limit is defined as the smallest amount (or lowest concentration) of the analyte that can be distinguished from the background noise of a blank matrix sample. The detection limit is calculated using Equation (1) specified in the National Standard GB/T 27404—2008 “Laboratory Quality Control Specifications for Food Physicochemical Testing” [29]. The linear range is determined based on the recovery experiments with spiked blank matrix samples.

### 4.2. Sample Pretreatment and Determination of AFB_1_ Content

#### 4.2.1. Sample Pretreatment

Accurately weigh 5.0 g of yellow rice wine using an electronic balance (model CP4102, Ohaus Instruments (Changzhou) Co., Ltd, Changzhou, Jiangsu Province, China) and place it into a 50 mL centrifuge tube. Add 16 mL methanol-water solution (78 parts methanol and 22 parts water) and mix thoroughly in a shaker (KB5010, Haikou Qilinbei Instrument Manufacturing Co., Ltd., Haikou, China) for 15 min. The motion type of the shaker is circular oscillation (gyratory oscillation), with an amplitude (radius of gyration) of 15 mm and an oscillation frequency of 240 revolutions per minute. Perform high-speed centrifugation (model 5810R, Eppendorf, with the oscillation time 15 min and the centrifugation speed 6000 r/min). Collect 1.0 mL of the supernatant, add 3.0 mL of sample diluent, and mix well using a vortex mixer. Filter the mixture through an organic filter membrane to obtain the test solution, which is then kept on standby.

#### 4.2.2. Determination of AFB_1_ Content

Open the sample cup and add 150 µL of the prepared test sample. Ensure complete dissolution and mix thoroughly. Place the sample cup in the heater and incubate at 34 °C for 6.4 min. Remove a test strip from the test strip tube and insert it into the sample cup with the arrow pointing downward to allow for chromatography. Follow the operating steps of the time-resolved fluorescence rapid detection instrument for AFB_1_ to perform hardware self-inspection. After chromatography, remove the test strip and dry the lower end of the strip. Within 2 min, use the time-resolved fluorescence reader to obtain the signal value for the determination of AFB_1_ content.

Note: The time-resolved fluorescence rapid detection instrument for aflatoxin, as well as the anti-aflatoxin B_1_ antibodies, incubator, sample cups, and other accessories, were developed by the team members in the preliminary stage [25].

### 4.3. Experimental Designs

#### 4.3.1. Single-Factor Experiment

The study investigates the effects of different methanol-water volume fractions (50%, 60%, 70%, 80%, 90%), sample-to-extraction solvent ratios (1:2, 1:3, 1:4, 1:5, 1:6 (mL:mL)), oscillation times (10, 15, 20, 25, 30 min), centrifugation speeds (4000, 5000, 6000, 7000, 8000 r/min), centrifugation times (2, 3, 4, 5, 6 min), heating temperatures (25, 28, 31, 34, 37 °C), and heating times (2, 4, 6, 8, 10 min) on the recovery rate of AFB_1_ in yellow rice wine. Each experiment was repeated three times, and the average value was taken.

#### 4.3.2. Plackett-Burman Design

The Plackett-Burman design is an efficient method for identifying significant factors among a large number of variables [30]. In this study, Design-Expert Version 13 (Stat-Ease Ine., Minneapolis, MN, USA) was employed to screen for important variables that significantly affect the recovery rate of AFB_1_.

#### 4.3.3. Path of Steepest Ascent

The steepest ascent method is a procedure for moving along the maximum increase in the response [31,32]. The direction of the steepest ascent represents the trajectory along which the response experiences the most rapid increase through the adjustment (either increase or decrease) of the values of significant factors. The zero-level settings of the Plackett-Burman design were designated as the starting point for the steepest ascent path. The step size along this path was determined by integrating the estimated coefficients from Equation (5) with practical experience. Experiments were conducted sequentially along the steepest ascent path until the response ceased to increase. This terminating point, which is likely in the vicinity of the optimal point, was then selected as the center point for the Central Composite Design (CCD) [33].

#### 4.3.4. Response Surface Methodology

The optimal levels of the significant factors and the interactions of these variables on recovery rate were analyzed by CCD. In this study, Design-Expert Version 13 (Stat-Ease Ine., Minneapolis, MN, USA) was used for designing experiments as well as for regression and graphical analysis of the experimental data obtained. Analysis of variance (ANOVA) was employed to assess the significance of the model and regression coefficients. The goodness-of-fit of the polynomial equation was evaluated using the coefficient of determination (R^2^), and its statistical significance was verified through the Fischer’s F-test. The significance of individual regression coefficients was examined via the Student’s t-test. Response surface plots and contour plots of the model-predicted responses were used to explore the interactive relationships among the significant variables.

### 4.4. Validation of the Model

#### 4.4.1. Intra-Assay and Inter-Assay Precision of the Method

A blank matrix sample was added with a recovery test to obtain the precision of this method. Add AFB_1_ standard working solution into the blank matrix samples of yellow rice wine to attain final concentrations of AFB_1_ at 1, 5, and 10 µg·kg^−1^, respectively. Using AFB_1_ time-resolution fluorescence immunochromatography test strips, calculate the average addition recovery rate and relative standard deviation. The intra-assay precision was determined from the same operator with the assay repeated six times, and the inter-assay precision was determined from different operators for six successive days, respectively.

#### 4.4.2. Comparison of Result Between TRFIA and HPLC

Ten naturally contaminated yellow rice wine samples and 10 blank yellow rice wine matrix samples spiked with random concentrations were selected. These samples were analyzed using TRFIA and HPLC-PHRED-FLD (GB 5009.22—2016), respectively, to evaluate the consistency between the two methods.

## Figures and Tables

**Table 1:** Levels of the variables tested in Plackett-Burman design.

		Experimental Value	
Variable	Symbol	Low (−1)	High (+1)
Methanol-water volume fraction (%)	A	60	80
Sample to extraction solvent ratio	B	1:2	1:4
Oscillation time (min)	C	12	18
Centrifugal speed (r/min)	D	5000	7000
Centrifugal time (min)	E	3	5
Heating temperature (°C)	F	30	37
Heating time (min)	G	4	8
Dummy1	H	−1	1
Dummy2	I	−1	1
Dummy3	J	−1	1
Dummy4	K	−1	1

**Table 2:** Plackett-Burman design of variables (in coded levels) with AFB_1_ as response.

Run Order	Experimental Value											Recovery Rate (%)
	A	B	C	D	E	F	G	H	I	J	K	
1	−1	1	1	−1	1	1	1	−1	−1	−1	1	88.3
2	−1	−1	−1	−1	−1	−1	−1	−1	−1	−1	−1	72.9
3	1	−1	1	1	−1	1	1	1	−1	−1	−1	94.5
4	−1	1	1	1	−1	−1	−1	1	−1	1	1	72.5
5	−1	1	−1	1	1	−1	1	1	1	−1	−1	84.6
6	−1	−1	−1	1	−1	1	1	−1	1	1	1	88.1
7	1	1	−1	−1	−1	1	−1	1	1	−1	1	94.6
8	1	−1	1	1	1	−1	−1	−1	1	−1	1	81.6
9	1	1	−1	1	1	1	−1	−1	−1	1	−1	94.8
10	1	−1	−1	−1	1	−1	1	1	−1	1	1	88.3
11	−1	1	1	−1	1	1	−1	1	1	1	−1	81.2
12	1	−1	1	−1	−1	−1	1	−1	1	1	−1	90.5

A–K are symbols shown in Table 1.

**Table 3:** Effects of the variables and statistical analysis of the Plackett-Burman design.

	Coefficient	Sum of Squares	Degrees of Freedom	Mean Square	F-Value	*p*-Value	
Model	85.99	647.61	7	92.52	38.97	0.0016	**
A	4.73	267.91	1	267.91	112.84	0.0004	***
B	1.56	29.14	1	29.14	12.27	0.0248	*
C	−1.23	18.01	1	18.01	7.58	0.0512	
D	0.025	0.0075	1	0.0075	0.0032	0.9579	
E	0.475	2.71	1	2.71	1.14	0.3457	
F	4.26	217.60	1	217.60	91.65	0.0007	***
G	3.06	112.24	1	112.24	47.28	0.0023	**
Residual		9.50	4	2.37			
Correlation Total		657.11	11				

Variables with a confidence level exceeding 95% are considered significant parameters (*), those with a confidence level exceeding 99% are considered highly significant parameters (**), and those with a confidence level exceeding 99.9% are considered extremely significant parameters (***).

**Table 4:** Experimental design and response value of path of steepest ascent.

Items	X_1_	X_2_	X_3_	X_4_	Recovery Rate (%)
No. 1. Base point (zero level in Plackett-Burman design)	70	1:3	33.5	6	
No. 2. Origin step unit (range of unity level)	10	1	3.5	2	
No. 3. Slope (estimated coefficient ratio from Equation (5)	+4.73	+1.56	+4.26	+3.06	
No. 4. Correspondent range = 2 × 3	47.3	1.56	14.91	7.12	
No. 5. New step unit = (4) × 0.1 ^a^	4.73	0.156	1.49	0.712	
No. 6. New step unit with a decimal	5.0	0.2	1.5	1.0	
Experiment No. 1	65	1:2.8	30	4	71.8
Experiment No. 2	70	1:3.0	31.5	5	79.4
Experiment No. 3	75	1:3.2	33	6	96.1
Experiment No. 4	80	1:3.4	35.5	7	90.1
Experiment No. 5	85	1:3.6	37	8	82.2

0.1 ^a^ is a factor determined by experiment based on process knowledge and is appropriate in this experiment.

**Table 5:** Levels of the variables tested in the CCD.

Variable	Symbol	Coded Level				
		−2	−1	0	1	2
Methanol-water volume fraction	X_1_	65	70	75	80	85
Sample to extraction solvent ratio	X_2_	1:2.8	1:3.0	1:3.2	1:3.4	1:3.6
Heating temperature	X_3_	29	31	33	35	37
Heating time	X_4_	4	5	6	7	8

**Table 6:** Experimental design and results of CCD.

Run Order	Code Level				Recovery Rate (%)	Run Order	Code Level				Recovery Rate (%)
	X_1_	X_2_	X_3_	X_4_			X_1_	X_2_	X_3_	X_4_	
1	1	1	−1	−1	83.2	16	−1	1	1	1	79.5
2	0	0	0	0	95.7	17	0	0	0	0	96.7
3	0	0	0	2	83.1	18	0	0	−2	0	80.2
4	1	1	−1	1	88.6	19	−1	1	−1	1	78.9
5	−2	0	0	0	78.5	20	1	−1	−1	1	82.3
6	0	2	0	0	86.3	21	1	1	1	1	92.1
7	0	0	0	−2	75.5	22	−1	1	1	−1	80.2
8	−1	−1	−1	1	76.6	23	2	0	0	0	88.7
9	1	1	1	−1	83.3	24	0	0	0	0	96.4
10	−1	−1	1	1	82.9	25	−1	−1	1	−1	80.8
11	1	−1	1	1	92.6	26	0	0	0	0	94.9
12	0	0	2	0	87.9	27	0	−2	0	0	82.6
13	1	−1	−1	−1	73.3	28	0	0	0	0	95.1
14	−1	1	−1	−1	81.2	29	0	0	0	0	95.2
15	−1	−1	−1	−1	78.6	30	1	−1	1	−1	84.2

**Table 7:** ANOVA for response surface quadratic model.

Source	Sum of Squares	Degrees of Freedom	Mean Square	F-Value	*p*-Value	
Model	1371.77	14	97.98	121.96	<0.0001	significant
X_1_-X_1_	156.57	1	156.57	194.89	<0.0001	
X_2_-X_2_	22.23	1	22.23	27.67	<0.0001	
X_3_-X_3_	97.20	1	97.20	120.99	<0.0001	
X_4_-X_4_	80.30	1	80.30	99.95	<0.0001	
X_1_X_2_	12.08	1	12.08	15.03	0.0015	
X_1_X_3_	17.43	1	17.43	21.70	0.0003	
X_1_X_4_	74.39	1	74.39	92.60	<0.0001	
X_2_X_3_	43.89	1	43.89	54.63	<0.0001	
X_2_X_4_	2.48	1	2.48	3.09	0.0993	
X_3_X_4_	4.52	1	4.52	5.62	0.0316	
X_1_X_1_	262.35	1	262.35	326.55	<0.0001	
X_2_X_2_	227.54	1	227.54	283.22	<0.0001	
X_3_X_3_	243.61	1	243.61	303.23	<0.0001	
X_4_X_4_	476.43	1	476.43	593.02	<0.0001	
Residual	12.05	15	0.8034			
Lack of fit	9.32	10	0.9318	1.70	0.2893	not significant
Pure error	2.73	5	0.5467			
Correlation total	1383.82	29				

R^2^ = 0.9918; pred-R^2^ = 0.9832; adj-R^2^ = 0.9584.

**Table 8:** Results of intra-assay precision (n = 6).

Spiked Level (μg·kg^−1^)	Average Finding (μg·kg^−1^)	Recovery Rate (%)	Standard Deviation (μg·kg^−1^)	RSD (%)
1	0.879	87.9	0.0550	6.26
5	4.885	97.7	0.2345	4.80
10	10.569	105.7	0.4734	4.48

**Table 9:** Results of inter-assay precision (n = 6).

Spiked Level (μg·kg^−1^)	Average Finding (μg·kg^−1^)	Recovery Rate (%)	Standard Deviation (μg·kg^−1^)	RSD (%)
1	0.859	85.9	0.0761	8.86
5	4.760	95.2	0.3380	7.10
10	9.980	99.8	0.6088	6.10

**Table 10:** Comparison of detection results for natural samples between TRFIA and HPLC.

Sample Number	Finding by TRFIA (μg·kg^−1^)	Finding by HPLC (μg·kg^−1^)	Relative Error (%)
Sample 1	1.646	1.543	6.68
Sample 2	N.D.	N.D.	/
Sample 3	N.D.	0.157	/
Sample 4	2.105	2.185	3.66
Sample 5	6.710	6.910	2.89
Sample 6	N.D.	N.D.	/
Sample 7	1.200	1.330	7.69
Sample 8	N.D.	N.D.	/
Sample 9	N.D.	0.09	/
Sample 10	0.612	0.684	10.53

N.D.: No detected.

**Table 11:** Performance comparison of TRFIA, ELISA, HPLC, and LC-MS/MS.

Parameters	TRFIA	ELISA [12,24]	HPLC [24]	LC-MS/MS [24,26,27]
Detection limit	0.3 μg/L	0.05–1 μg/L	0.02–0.03 μg/L	0.03–0.5 μg/L
Limit of quantitation	0.8 μg/L	0.1–3 μg/L	0.05–0.1 μg/L	0.1–0.25 μg/L
Analysis time	30–60 min	45–90 min	2–3 h	2–3 h
Specificity	high	low	extremely high	extremely high

## Data Availability

The original contributions presented in this study are included in the article. Further inquiries can be directed to the corresponding authors.
